# Life Course Adversity and Early Retirement Due to Poor Health

**DOI:** 10.1093/geroni/igab046.102

**Published:** 2021-12-17

**Authors:** Amanda Sonnega, Brooke Helppie-McFall, Haena Lee

**Affiliations:** 1 University of Michigan, Ann Arbor, Michigan, United States; 2 University of Southern California, Los Angeles, California, United States

## Abstract

The relationship between life adversity and physical and mental health is well documented. The present research investigates life course adversity and early retirement due to poor health. Data are from the Health and Retirement Study, including the Life History Mail Survey (LHMS), HRS core surveys, and HRS Psychosocial Leave-Behind surveys. We create measures of childhood financial and social adversity and young-mid adulthood financial and social adversity. Early retirement is defined as self-report of “full” retirement between age 50 and 62. We use a Cox proportional hazards model competing risk framework comparing early retirement when poor health is a major reason for retirement with early retirement for any other reason. Models include covariates for age, gender, marital status, cohort, log household income, and log household wealth. Childhood financial adversity and homelessness in young-mid adulthood increases the instantaneous hazard of early retirement due to poor health.

